# Multi-targeted, NOT gated CAR-T cells as a strategy to protect normal lineages for blood cancer therapy

**DOI:** 10.3389/fimmu.2025.1493329

**Published:** 2025-03-21

**Authors:** Breanna DiAndreth, Pavlo A. Nesterenko, Aaron G. Winters, Aaron D. Flynn, Claudia A. Jette, Vasantika Suryawanshi, Sanam Shafaattalab, Sara Martire, Mark Daris, Elizabeth Moore, Ryan Elshimali, Tanveer Gill, Timothy P. Riley, Sara Miller, Chawita Netirojjanakul, Agnes E. Hamburger, Alexander Kamb

**Affiliations:** A2 Biotherapeutics Discovery Research, Agoura Hills, CA, United States

**Keywords:** CAR-T cells, CD33, CD16, SPN, AML, LIR-1 (LILRB1), logic gate, blood cancer

## Abstract

**Introduction:**

Despite advances in treatment of blood cancers, several—including acute myeloid leukemia (AML)—continue to be recalcitrant. Cell therapies based on chimeric antigen receptors (CARs) have emerged as promising approaches for blood cancers. However, current CAR-T treatments suffer from on-target, off-tumor toxicity, because most familiar blood cancer targets are also expressed in normal lineages. In addition, they face the common problem of relapse due to target-antigen loss. Cell therapeutics engineered to integrate more than one signal, often called logic-gated cells, can in principle achieve greater selectivity for tumors.

**Methods:**

We applied such a technology, a NOT gated system called Tmod™ that is being developed to treat solid-tumor patients, to the problem of therapeutic selectivity for blood cancer cells.

**Results:**

Here we show that Tmod cells can be designed to target 2-4 antigens to provide different practical and conceptual options for a blood cancer therapy: (i) mono- and bispecific activating receptors that target CD33, a well-known AML antigen expressed on the majority of AML tumors (as well as healthy myeloid cells) and CD43 (SPN), an antigen expressed on many hematopoietic cancers (and normal blood lineages); and (ii) mono- and bispecific inhibitory receptors that target CD16b (FCGR3B) and CLEC9A, antigens expressed on key normal blood cells but not on most blood cancers.

**Discussion:**

These results further demonstrate the robust modularity of the Tmod system and generalize the Tmod approach beyond solid tumors.

## 1 Introduction

Certain blood cancers such as AML are deadly diseases, with no broadly effective therapies other than chemotherapy preconditioning combined with stem cell transplant (1, 2). One of the fundamental challenges is the lack of genes expressed uniquely in AML and other blood cancers that can be exploited as molecular targets for selective elimination of malignant cells (3, 4). Most (if not all) AML molecular targets under consideration are also expressed in subsets of normal lineages. A good example is CD33, a lectin that is expressed on >90% of AML tumors but also on normal immune cells, especially myeloid cells such as monocytes, promyelocytes and neutrophils (5–8). Despite the relative appeal of CD33 as an AML tumor-associated antigen (TAA), therapeutics targeting CD33 have substantial clinical toxicity, presumably caused at least in part by expression on healthy myeloid cells (9–11). For instance, an FDA-approved antibody-drug conjugate (Mylotarg™) has serious dose-limiting toxicity that limits its use and efficacy, some of which is likely caused by on-target toxicity (12–15). CD33-directed T cell engagers have encountered similar problems in the clinic, with the additional challenge of cytokine release syndrome (CRS), a potentially serious immune overreaction caused by antigen expression on normal cells (16).

Although cell therapies have emerged as a powerful new weapon against certain types of blood cancer including Non Hodgkin’s lymphoma (NHL) and multiple myeloma (17, 18), so far they have not demonstrated the same success in AML (19). In contrast to NHL and certain other blood cancers, AML does not possess any genes that are expressed: (i) in the large majority of tumors indicated for treatment; and (ii) only in normal cells that are not required for survival in the short term (e.g., B cells) (7, 8). Indeed, there is little to suggest that TAA-directed AML therapeutics, including CD33 CAR-Ts, can avoid the toxicities caused by loss of key normal immune cells. This poses immediate safety issues and longer-term risk of infection (20). Though the precise cause of toxicities in individual patients can be complex and difficult to elucidate, CD33-directed investigational cell therapies can be severely toxic, and potentially fatal (21). This places drug developers in a quandary: How can toxicities be mitigated while maintaining the advantages of TAAs such as CD33?

**Figure 1:**
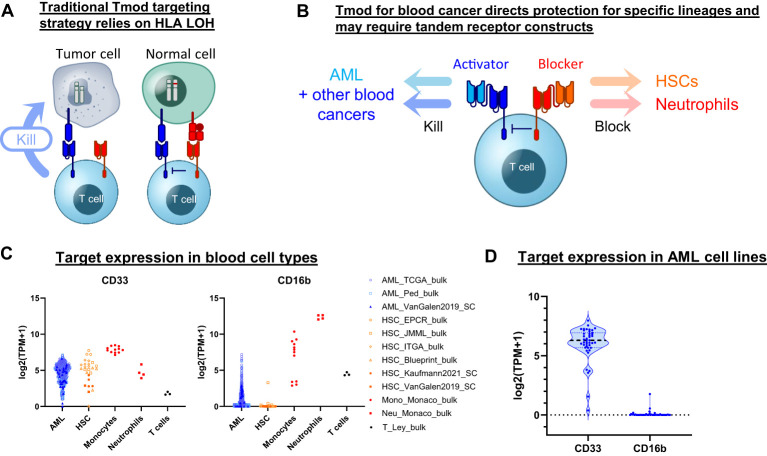
Tmod can be adapted for blood cancer. **(A)** Diagram for Tmod system showing the two receptors that comprise the NOT gate targeting HLA loss of heterozygosity (LOH) in solid tumors. **(B)** Diagram for Tmod utilizing tandem receptors for blood cancer. **(C)** CD33 and CD16b mRNA expression in primary AML and healthy blood cells including T cells, neutrophils, monocytes, and hematopoietic stem cells (HSC) (data from sources shown; see **Supplementary Table 1**). **(D)** mRNA expression of targets in AML cell lines (n=43; DepMap).

One possibility involves cell therapeutics that respond to more than one antigen to achieve better selectivity toward AML cells. An interesting option is to engineer a NOT gate in immune cells to protect normal CD33-expressing cells without compromising AML cytotoxicity (22, 23). The best studied NOT gate is Tmod, a system that has demonstrated robust activity in multiple preclinical settings (24–26). Tmod is being developed to treat patients whose tumors have lost one or both alleles at the HLA locus via deletion (25, 27). This system employs T cells engineered to express an activating receptor (i.e. CAR or TCR) paired with a “blocker” derived from the LIR-1 (LILRB1) inhibitory receptor, the natural ligands of which are HLA-I molecules (28) (**Figure 1A**). Tmod therapeutic constructs have been shown to work in many tumor models of HLA deletion.

Here we describe a Tmod construct designed to improve the safety of CD33 targeted therapeutics. The engineered Tmod cells respond to CD33 only if a second antigen, CD16b, is absent. Because CD16b is expressed on normal myeloid lineages and not on AML, this approach offers the possibility of mitigating myeloid compartment depletion and CRS by specifically blocking healthy-cell-mediated activation of the CD33 | CD16b Tmod cells by normal myeloid cells. We build on this construct by adding OR-gating capability through additional ligand-binding domains to broaden the potential applicability of Tmod beyond monospecific AML designs. These designs include bispecific receptors that target 2 activator antigens (CD33 and SPN) and 2 blocker antigens (CD16b and CLEC9A) (**Figure 1B**). The results support further investigation of the Tmod approach to improve the therapeutic window of future blood cancer medicines and provide further proofs of concept for the use of non-HLA-I proteins as blocker antigens for Tmod constructs.

## 2 Results

### 2.1 CD16b is an attractive blocker antigen for CD33 CAR-Ts

We sought to identify a blocker antigen compatible with Tmod constructs that incorporate a CD33 CAR activator. A bioinformatic search of RNA-Seq databases (29–40)(**Supplementary Table 1**) revealed that CD16b (*FCGR3B*) expression not only corresponds with CD33 expression in neutrophils but also is largely absent in primary AML specimens (**Figure 1C**). CD16b, first identified as Human Neutrophil Antigen-1, is a glycosylphosphatidylinositol-anchored protein that functions as a low-affinity receptor for the Fc region of IgG, with high surface expression limited to granulocytes (41). The absence of *FCGR3B* expression in AML is not the consequence of genetic deletion, but rather the epigenetic state of the malignant cell (**Supplementary Figure 1**). Low expression of *FCGR3B* is also observed in the large majority of AML cell lines, suggesting that its transcriptional off-state is robustly maintained in cell culture—much as the CD33 on-state is maintained (**Figure 1D**). Thus, based on expression profile alone, CD16b is an attractive blocker antigen for a Tmod construct directed at CD33.

### 2.2 Generation of CD16 scFvs that function effectively in CD33 | CD16 Tmod constructs

To create CD33 | CD16b Tmod constructs, we began with a set of 4 CD33 CARs (CAR1-4) that were sensitive enough to respond to CD33 in AML-derived cell lines and two other cells lines (HeLa and K562) transfected with CD33 mRNA (**Supplementary Figure 2**; see Methods). We combined CAR1 with a blocker that uses an scFv derived from a commercially available monoclonal antibody (mAb; 3G8) (42) that binds both human CD16 paralogs (CD16a and CD16b) with high affinity. CD33 Tmod constructs using this CD16-directed scFv demonstrated selective response in both Jurkat reporter assays (**Supplementary Figure 3A**) and in primary T cell cytotoxicity assays (**Supplementary Figure 3B**). These results supported the feasibility of targeting CD16 via CD33 | CD16 Tmod constructs. However, because CD16a is expressed in a higher percentage of AML samples than CD16b (**Supplementary Figure 4**), a search for CD16b-specific binders was undertaken.

**Figure 2:**
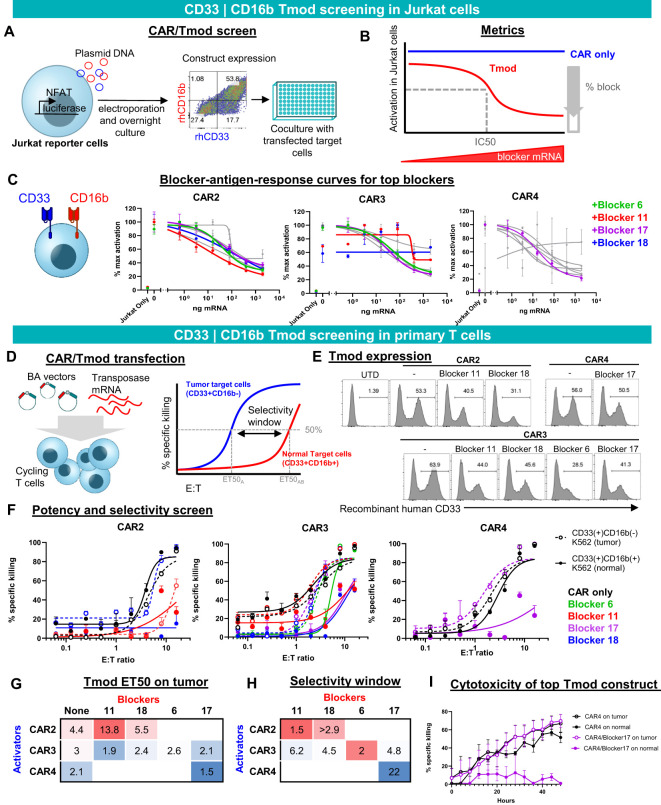
CD33 | CD16b Tmod functions robustly in Jurkat and primary T cells. **(A)** Diagram of functional screen in Jurkat reporter cell line cocultured with K562 target cells transfected with different amounts of CD16b mRNA. Tmod transgene expression in Jurkat cells was detected by staining with recombinant human (rh) CD16b and CD33. **(B)** Diagram of functional parameters estimated from the Jurkat cell assay data. **(C)** Functional readout from 8-point mRNA titration curves. Three CARs combined with 4 blockers, that were selected for further analysis, are shown in color. Data are shown as mean ± standard deviation of technical replicates (n=2), normalized to each sample’s maximum activation. **(D)** Left, diagram of non-viral construct-screening in primary T cells using PiggyBac transposase and single vectors (BA vectors). Right, metrics used to quantify the potency and selectivity of the Tmod pair. **(E)** Flow cytometry analysis of stable integrants via staining with labeled recombinant human CD33 (see Methods). **(E, F)** T cell cytotoxicity curves generated from GFP signal at 48 hour time point with each well normalized to the zero time point. Tumor (CD33(+)CD16(-)) target-cell curves are shown with dashed lines and “normal” (CD33(+)CD16(+)) target-cell curves with solid lines. Black lines are CAR constructs and colored lines are Tmod constructs. Tumor cells are K562 cells engineered with CD33 and normal cells are K562 cells engineered to overexpress CD33 and CD16b. Data are shown as mean ± standard deviation of technical replicates (n=3). **(G)** Potency calculated as ET50 of Tmod cells cocultured with tumor cells. **(H)** Selectivity ratios are calculated as ET50 on normal cells divided by ET50 on tumor cells. **(I)** Kinetic cytotoxicity analysis of the most selective and potent construct compared to the CAR-T. GFP(+) area was used as proxy for target cell viability. Data are shown as mean ± standard deviation of technical replicates (n=3).

Despite the high similarity of the two CD16 paralogs (98% amino acid identity), a mouse immunization campaign, using soluble recombinant CD16b protein as the antigen, yielded a panel of CD16b-selective antibodies (**Supplementary Figure 5A**). Selectivity was confirmed in ELISA assays and via flow cytometry with engineered K562 cells that overexpress either CD16a or CD16b (**Supplementary Figure 5B,C**). Diverse VH and VL sequences were recovered from 32 CD16b-selective antibodies (**Supplementary Figure 5D**; see Methods). The VH and VL sequences were used to construct scFvs and fused to the LIR-1 (LILRB1) backbone to create blocker receptors. Tmod constructs encoding the CD33 CARs and CD16b blockers were tested in assays using engineered Jurkat cells cocultured with K562 target cells that overexpressed CD33. mRNA titration was used to estimate ligand-dependent inhibition (**Figures 2A, B**). The initial screen focused on a single CD33 CAR as the activator component of Tmod to measure percent-inhibition in K562 cells. The 10 top-ranked blockers in this assay were selected based on percent blocking and IC50 for further analysis (**Supplementary Figure 6A**). These 10 blockers were screened independently with 3 additional CD33 CARs (4 total) to identify the most robust and modular blockers (**Supplementary Figure 6B**). Finally, the top 6 blockers from this assay, plus the 3G8 clone benchmark, were combined for further testing with a set of 3 top CARs. These CD33 | CD16b Tmod combinations were profiled using 8-point mRNA dose-response titrations. Fitted sigmoid curves allowed selection of the CD33 | CD16b Tmod receptor pairs with optimal performance, based on a combination of high sensitivity (low IC50) and high maximum percent inhibition (**Figure 2C**, **Supplementary Figure 6C**). From this analysis, 9 pairs with varied functional profiles were advanced for further testing in T cells.

### 2.3 CD33 | CD16 Tmod cells display potent, selective cytotoxicity *in vitro*

To create constructs suitable for expression in primary human T cells, we generated PiggyBac vectors with inserts that produce a single transcript encoding bicistronic CD33 | CD16b Tmod. We term these BA vectors because the blocker is encoded upstream of the activator (**Figure 2D**), which helps to prevent activator-only expression (**Supplementary Figure 8A**). Of 9 pairs that were chosen to advance to the primary T cell stage, 7 expressed well as measured by staining with labeled CD33 soluble protein. Though some variability in the percent Tmod(+) cells was observed, the expression system consistently produced 30-50% Tmod(+) cells, depending on the CD33 | CD16b pair (**Figure 2E**).

We focused on two key parameters of Tmod function in cytotoxicity assays: potency and selectivity (**Figure 2D**, right). To measure these parameters, E:T titration experiments were performed using two variants of K562 as the target cells: (i) CD33(+)CD16b(-) cells that overexpress CD33, intended to represent tumor cells; and (ii) CD33(+)CD16b(+) cells that overexpress both CD33 and CD16b, intended to model normal myeloid cells in this experiment (**Figure 2F**). Potency was estimated by the E:T ratio at 50% maximum killing (ET50). As a rough gauge of potency, ET50s of Tmod cells cocultured with CD33(+)CD16(-) target cells were compared to the corresponding CAR T cells without the blocker. A lead pair (CAR4 | Blocker17) was selected for further study based on potency of the Tmod cells similar to the CAR control (**Figure 2G**).

We defined selectivity as a ratio of ET50 using surrogate normal CD33(+)CD16b(+) cells divided by ET50 using tumor CD33(+)CD16b(-) cells. The construct that had the best potency (CAR4/Blocker17) also had the best selectivity window (**Figure 2H**). This CD33 | CD16b Tmod construct also displayed robust on-target activity and good normal cell selectivity in kinetic assays of cytotoxicity (**Figure 2I**). Thus, the CAR4 | Blocker17 construct displayed several desirable features of a CD33 | CD16b Tmod candidate: high potency (low ET50) and high selectivity window (22x).

### 2.4 CD33 | CD16 Tmod cells selectivity kill CD33(+)CD16(-) AML cells *in vivo*

**Figure 3:**
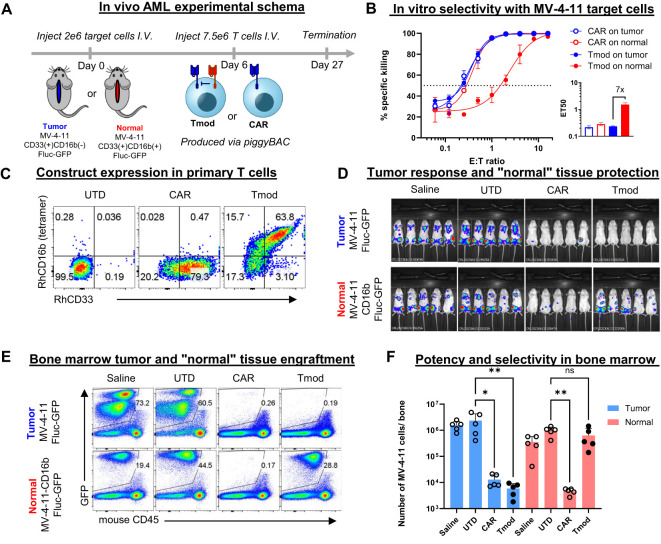
CD33 | CD16b Tmod cells selectively kill tumor but not “normal” cells *in vivo*. **(A)** Schema for ***in vivo*** experiment. 2 million MV-4-11 AML cells or MV-4-11 cells that overexpress CD16b were injected into NSG-SGM3 mice and 6 days later 7.5 million T cells were injected. **(B)** Selectivity *in vitro* using MV-4-11 cells. Surrogate normal cells were generated by overexpression of CD16b in the AML cells. E:T cytotoxicity curves were generated from firefly luciferase bioluminescence at 48 hours. Data are shown as mean ± standard deviation of technical replicates (n=3). Inset: ET50 values of depicted curves. Data shown are interpolated values with 95% CI. **(C)** Flow cytometry analysis of construct expression by staining with labeled recombinant human CD16b and CD33. **(D)** Bioluminescence imaging (BLI) at 20 days post target-cell injection. **(E)** Flow cytometry analysis of MV-4-11 cells in the bone marrow 27 days post target-cell injection. **(F)** Quantification of data shown in panel. **(E)** Statistics were calculated using a non-parametric Kruskal-Wallis *H* test; *: 0.01 < adjusted p < 0.05; **: adjusted p value < 0.01; ns: not significant (adjusted p > 0.05).

To test CD33 | CD16b Tmod cells *in vivo*, we utilized a xenograft model based on growth of the AML line MV-4-11 in NSG mice after intravenous infusion of tumor cells (**Figure 3A**). MV-4-11 cells express endogenous CD33 at levels similar to primary AML samples and within the sensitivity range of our lead CAR (**Supplementary Figure 7**). Because these cells do not express CD16b, we engineered a variant line to model normal cells by exogenous expression of CD16b (**Supplementary Figure 8B**). Both variants were further modified to express firefly luciferase and green fluorescent protein (GFP) for detection. The pair of MV-4-11 cell lines, when cocultured *in vitro* with a titration of effector CD33 | CD16b Tmod cells, elicited the expected pattern of cytotoxicity; i.e., a selectivity window of 7x (**Figure 3B**). CD33 | CD16b Tmod cells generated via PiggyBac and cultured for 31 days showed high expression of the Tmod components (**Figure 3C**).

Six days after the grafts were introduced, T cells were injected intravenously and MV-4-11 cells were monitored by BLI (**Figure 3A**). As observed *in vitro*, CD33 | CD16b Tmod cells killed the CD33(+)CD16b(-) tumor cells but not the CD33(+)CD16b(+) variant line *in vivo* (**Figure 3D**). As an independent readout of graft cell number, bone marrow was isolated from the mice at the end of the study (day 27 post MV-4-11 graft injection) and cells were analyzed by flow cytometry. In mice bearing CD33(+)CD16b(+) surrogate normal cells, numerous GFP(+) MV-4-11 cells were detected (**Figures 3E, F**). In contrast, very few graft cells were detected in mice from the CD33(+)CD16b(-) cohort, suggesting high selective killing *in vivo* of tumor *vs*. surrogate normal cells. Whereas CD33 CARs killed both MV-4-11 variant lines equally, CD33 | CD16b Tmod cells exhibited over 100x-fold increased killing of tumor cells compared to surrogate normal. Thus, *in vivo* AML tumor cells were killed by CD33 | CD16b Tmod cells while the surrogate normal cells, differing only by their expression of CD16b, were protected. These results provide a preclinical proof of concept for a potential CD33-targeted cell therapy gated by CD16b expression intended to eliminate AML cells in patients while sparing healthy myeloid cell types.

### 2.5 Tmod accommodates bispecific activators and blockers

**Figure 4:**
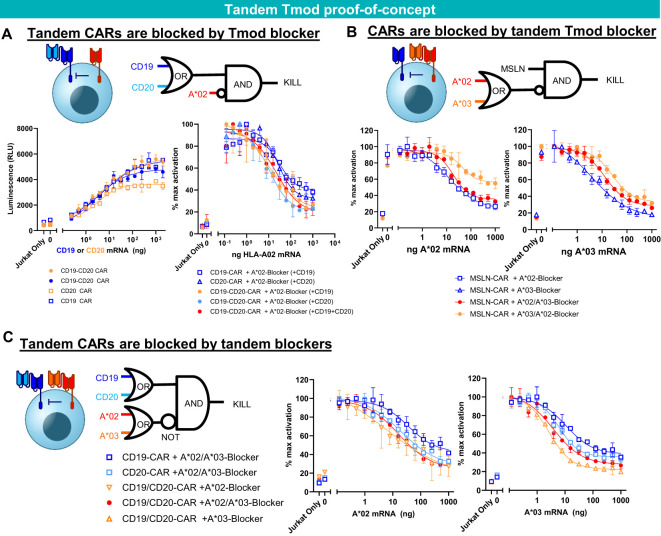
Tandem construct proof of concept. **(A)** A*02 blocker inhibits CD19-CD20 bispecific CAR. Jurkat functional readout from activator titration curves and blocker titration curves. **(B)** A*02-A*03 tandem blocker inhibits monospecific MSLN CAR. Jurkat (B2M KO) functional readout from blocker titration curves (either A*02 or A*03). **(C)** Tandem A*02-A*03 blocker inhibits tandem CD19-CD20 CAR. Jurkat (B2M KO) functional readout from blocker titration curves (either A*02 or A*03) with constant amounts of CD19 and CD20 mRNA. Data are shown as mean ± standard deviation of technical replicates (n=2).

To extend the potential utility of Tmod beyond monospecific designs, we first tested Tmod constructs with tandem activators and blockers using previously validated binders (**Figure 4**). As an activator, we tested a CD19-CD20 bispecific CAR (43) in Jurkat cell assays and demonstrated that it was effectively inhibited by a previously studied monospecific HLA-A*02 blocker (24, 25, 27) in a ligand-dependent fashion (**Figure 4A**). The HLA-A*02 blocker inhibited the tandem CAR as effectively as monospecific CARs. Importantly blocking of the tandem CAR was maintained with either one or both activator antigens present. Next, to explore bi-specific blockers of more familiar design, we showed that a mesothelin (MSLN) CAR (25) was blocked by a bispecific blocker composed of one scFv directed at HLA-A*02 and a second scFv in tandem directed at HLA-A*03 (25) (**Figure 4B**). Finally, these two bispecific receptors were combined in a single cell to demonstrate that the CD19-CD20 bispecific CAR was blocked by the tandem HLA-A*02-A*03 blocker (**Figure 4C**). Thus, the double-tandem Tmod NOT gate successfully integrated signals from 4 antigens—2 activator and 2 blocker antigens—to regulate effector cell activation and inhibition.

### 2.6 Multi-targeted Tmod applications for AML and other blood cancers

**Figure 5:**
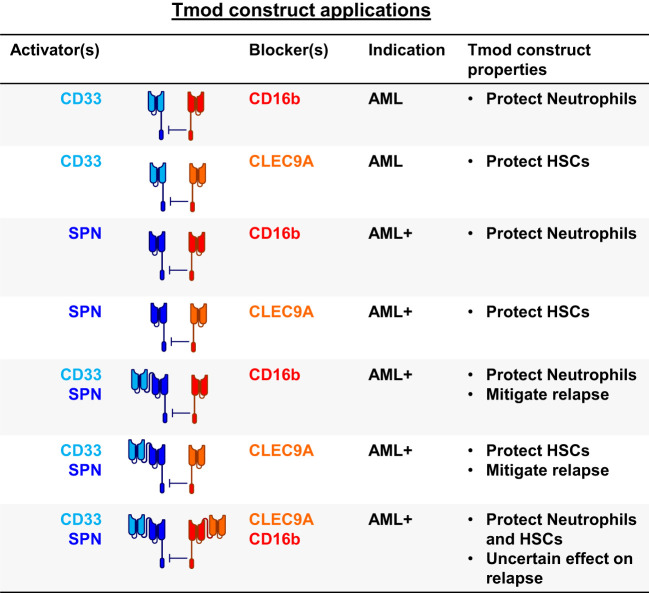
Blood cancer applications for Tmod constructs. SPN (CD43 protein); FCGR3B (CD16b protein); HSCs, hematopoietic stem cells; AML+ refers to blood cancers beyond AML.

**Figure 6:**
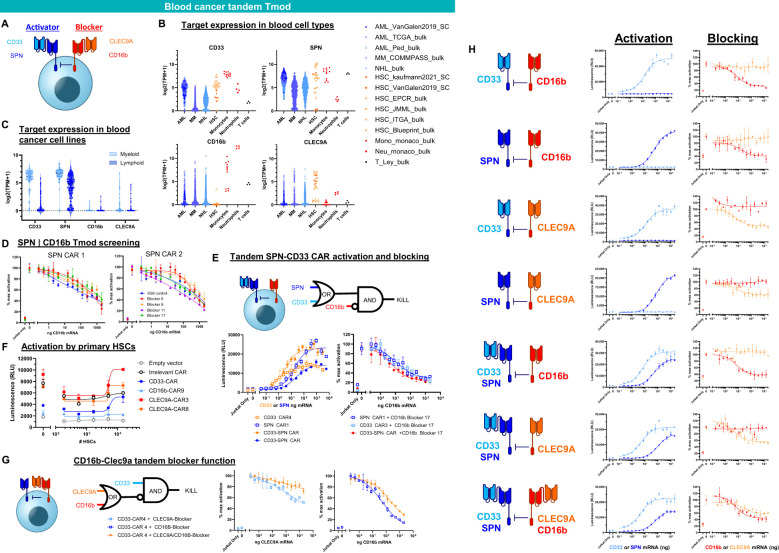
Tandem Tmod constructs for blood cancer. **(A)** Diagram of a Tmod cell with bispecific activator to target AML (CD33) and other blood cancers (SPN) and bispecific blocker to protect HSCs (CLEC9A) and neutrophils (CD16b). **(B)** Target expression in primary blood cancers and healthy blood cells (data from sources shown; see **Supplementary Table 1**). **(C)** Target expression in blood cancer cell lines (DepMap). **(D)** Jurkat cell (SPN KO) functional readout of SPN | CD16b Tmod with blocker titration curves. **(E)** Jurkat cell (SPN KO) functional readout of SPN-CD33 tandem CAR activation and blocking by CD16b blocker in the presence of SPN and CD33 antigens. **(F)** Jurkat functional readout of binders cloned as CARs with titration of primary HSCs. **(G)** Jurkat functional readout of CD33 CAR4 blocked by tandem CLEC9A-CD16b blocker. **(H)** Jurkat functional readout of CD33 and/or SPN monospecific or bispecific activators paired with CD16b and/or CLEC9A monospecific or bispecific blockers. Data are shown as mean ± standard deviation of technical replicates (n=2).

To incorporate these ideas and findings in the context of AML and other blood cancers, we tested a variety of bispecific constructs targeting CD33, CD16b and other blood-related antigens (**Figures 5**, **6A**). These were intended to target antigens expressed potentially on a broader population of blood cancers, while protecting other key normal lineages, primarily by sparing hematopoietic stem cells (HSCs). For AML, this approach also provided an opportunity to address AML relapse caused by CD33 antigen loss. To this end, we identified one additional activator antigen (SPN) and blocker antigen (CLEC9A) with expression profiles consistent with our aims (**Figure 6B, C**, **Supplementary Table 1**). SPN (CD43 or sialophorin) is a surface glycoprotein of poorly understood function highly expressed on most hematopoietic cells other than red blood cells (44). CLEC9A (CD370) is a C-type lectin known to be expressed on myeloid lineages (45) including HSCs (46, 47).

For SPN, two CARs with different scFvs directed at SPN were tested against 4 CD16b blockers (**Figure 6D**). All the constructs performed well (>50% block). One of the SPN CARs was converted to bispecific SPN-CD33 formats and tested with various binders in different orientations (**Supplementary Figure 9A**). The top-ranked 3 tandem CARs were paired with CD16b Blocker17 (**Supplementary Figure 9B**) and shown to regulate Jurkat-cell function effectively (see data from best-performing pair in **Figure 6E**).

For CLEC9A, novel CLEC9A binders were identified and screened as CARs (**Supplementary Figure 10**; see Methods). Two top-ranked binders were then tested for responsiveness to primary HSCs (**Figure 6F**). While controls and CD16b CAR-T cells did not show HSC-dependent activation, the CLEC9A CAR-Ts showed responses similar to a CD33 CAR-T, suggesting sufficient target expression and binder sensitivity for response. These top CLEC9A binders were then cloned as blockers and, in tandem with CD16b Blocker17 scFv, shown to function well (see best-performing construct in **Figure 6G**). Constructs that contained the tandem activators and blockers (CD33-SPN | CD16b-CLEC9A Tmod) expressed in Jurkat cells and functioned as expected from the behavior of the individual components; i.e., each scFv responded to antigen titration without interference from the others (**Figure 6H**). Together, these data showed that Tmod functions with tandem activators and blockers directed at novel non-HLA-I antigens relevant to blood cancer therapy.

## 3 Discussion

Tumor *vs*. normal selectivity is arguably the key problem in cancer therapy and many approaches have been developed to address it. These efforts include: (i) targeting critical functional dependencies in tumors such as HER2 in breast cancer and BCR-ABL in CML (48–51); (ii) targeting TAAs such as CD19 in NHL and BCMA in multiple myeloma (52, 53); (iii) targeting tumor-specific antigens such as HPV E7 and other neoantigens in solid tumors (54–58); (iv) protease-cleavable masked cytotoxic proteins designed to activate selectively in solid tumors (59); and (v) logic-gated cell therapies that respond to antigen profiles rather than single antigens (60–63).

The Tmod system was developed to address the problem of tumor *vs*. normal tissue selectivity via a NOT gate (26). The NOT gate conceptually allows a different set of antigens to be engaged to confer selectivity—those that are absent from the tumor but present in key normal tissues (26). In its original form, Tmod is intended to exploit a situation that arises in numerous cancer patients where solid tumors lose one HLA-I allele via loss of heterozygosity (LOH) in the founding clone of the tumor (24, 64). HLA LOH in these patients confers a genetically identifiable, homogeneous difference on the tumors that can in principle direct Tmod cells gated by, for example, HLA-A*02 to selectively kill tumor cells that express a TAA but have lost expression of HLA-A*02 (25, 27).

Here we extend this approach to tumor types such as AML that do not undergo HLA LOH. To do this, we have developed a blocker that targets a non-HLA-I molecule, in this case CD16b, and paired it with a CD33 CAR to create a CD33 | CD16b Tmod construct that selectively kills CD33(+)CD16b(-) AML cells, but spares isogenic cells that express CD16b, *in vitro* and *in vivo*. The expression profile of CD16b (very low in AML and high in myeloid cells) suggests that CD33 | CD16b constructs can create a therapeutic window in AML patients where CD33 is expressed on key normal tissues. In addition, though the cause of CRS is debated, it is possible that engagement of CD16b to block T cell activation by myeloid cells may mitigate the component of CRS that arises from reaction to CD33-expressing normal cells (65, 66).

Creation of a functional CD33 | CD16b Tmod construct required solution of two technical problems: (i) selection of a blocker antigen that is compatible with blocker function and the inverse of an AML TAA (i.e., with expression that is low in AML and high in normal blood cells); and (ii) identification of an scFv that can distinguish CD16b from its very close paralog, CD16a, which is expressed in a subset of AML malignancies and thus could limit efficacy. These challenges were overcome by a combination of antigen selection with screens for binding and function.

To improve the CD33 | CD16b construct and address matters that include relapse from single-antigen-loss in AML, blood cancers beyond AML, and protection of HSCs, we created proof-of-concept Tmod designs that incorporate 2 additional antigen-binding domains directed at SPN and CLEC9A placed in tandem with the CD33 scFv for the SPN activator and the CD16b scFv for the CLEC9A blocker. Others have shown that a NOT gate targeting endomucin (EMCN) to protect HSCs and a tandem CD33-FLT3 CAR to target AML cells functions in preclinical experiments (67). Here we extend these concepts to include tandem blockers with broader protection of normal cells and activators capable in principle of targeting blood cancers beyond AML, including NHL and multiple myeloma that express SPN.

It is not clear *a priori* which of the many possible multi-targeted designs are optimal for AML and other blood cancers (see **Figure 5**). Targeting 2 activator antigens should mitigate relapse, a common problem with CD19 CAR-Ts in NHL and all AML therapies. Indeed CD19-C20 bispecific CAR-Ts are under development in NHL for this purpose (68). The advantage of using a broadly expressed antigen such as SPN, whose expression is also maintained in most blood-cancer-derived cell lines, is to not only address relapse in AML but also potentially extend the product to patients with other blood cancers. These additional antigens compound the risk of on-target, off-tumor toxicity, challenges that may be addressed by multi-antigen-targeted blockers. However, depending on the propensities of tumors to up-regulate, rather than lose, expression of genes, such blockers may introduce an additional mechanism for tumor relapse. Note also that inclusion of activators that target antigens expressed in T cells (e.g., SPN) may necessitate other measures to reduce fratricide, e.g., inclusion of shRNA modules or gene-disruption to reduce/eliminate expression of the target antigen in the engineered T cells.

The flexibility and modularity of Tmod demonstrated here and in other publications support exploration of the Tmod technology in tumors that do not exhibit LOH. Although LIR-1 (LILRB1) evolved to specialize in HLA-I antigens (69), it is now clear that, providing certain design rules are followed, non-HLA-I antigens can also function as redirecting ligands for LIR-1 (70, 71). These results are important because many tumor types including blood tumors and certain solid tumors such as prostate carcinoma (see GISTIC database) do not exhibit high rates of HLA LOH. In addition, even among solid tumors that exhibit significant rates of LOH, a large segment (>75%) remains that do not have HLA LOH. Patients with these tumors require a different therapeutic approach. The demonstration that non-HLA-I antigens can robustly control CAR activation in Tmod cells suggests that it may be possible to exploit instances where antigen expression is absent in tumors for reasons other than HLA LOH.

## 4 Materials and methods

### 4.1 Tissue culture

All cell lines were purchased from ATCC, except for the NFAT-firefly luciferase reporter Jurkat cell line that was purchased from BPS bioscience (#60621). K562 cells were maintained in RPMI supplemented with 10% heat inactivated FBS and 1% penicillin-streptomycin. HeLa cells were maintained in EMEM with 10% FBS. Jurkat cells were cultured in RPMI supplemented with 10% heat-inactivated FBS, 1% penicillin-streptomycin and 0.4% geneticin to maintain reporter expression. MV-4-11 cells were cultured in RPMI with 10% heat-inactivated FBS and 1% penicillin-streptomycin.

PBMCs were obtained from STEMCELL. Enriched T cells were derived from Leukopaks apheresed from healthy donors (Charles River), followed by enrichment using CD56 depletion and CD4/CD8 enrichment using a CliniMACS Prodigy prior to freezing. T cells were cultured in X-VIVO15 (Lonza) supplemented with 5% human AB serum, 1% penicillin-streptomycin and 300 IU/ml of IL-2 (STEMCELL). For functional assays IL-2 was not included. For HSC experiments, human CD34(+) HSPCs derived from bone marrow were purchased from Charles River Laboratory. Cryopreserved cells were thawed and cultured in StemSpan SFEMII medium (STEMCELL) supplemented with StemSpan™ CC110 (STEMCELL).

### 4.2 Construct design and cloning

CAR and blocker constructs were synthesized (IDT) and cloned via Golden Gate assembly into lentivirus or PiggyBac transposon vectors containing flanking inverted terminal repeats for transposase recognition and insulators to prevent epigenetic silencing of transgene expression. CAR and blocker transgenes were designed as described previously (25, 72). Briefly, third-generation CARs were created by fusing nucleotide sequences encoding VL-(G4S)3GG-VH scFvs to sequences encoding a CD8a hinge, CD28 transmembrane domain, and CD28, 4-1BB, and CD3z intracellular domains. Blockers were generated by fusing VL-(G4S)3GG-VH scFvs to the hinge, transmembrane, and intracellular domains of LIR-1 (LILRB1). For tandem designs, additional scFvs were fused to these constructs using a (G4S)4 linker in Fv orientations as described in **Supplementary Figure 9**. The 2 receptors were cloned into single “BA” vectors with the blocker (B) in the first position, T2A cleavage site, followed by the CAR (A). When indicated, a puromycin resistance gene was included to enable puromycin-driven selection of stably integrated transgenes. Plasmid DNA maxipreps were performed by Aldevron. Cloned products were verified using Sanger sequencing (Azenta) or Nanopore sequencing (Primordium). Antigen mRNA was synthesized by *in vitro* transcription as previously described (25, 72).

### 4.3 Hybridoma IgG discovery

CD1 mice (n=5; Charles River) were immunized with either recombinant CD16b (NA2) (Acro Biosystems) or Clec9a-Fc (R&D) emulsified in Complete Freund’s Adjuvant (Thermo) and/or Sigma Adjuvant System (Sigma) according to a modified Repetitive Immunization at Multiple Sites (RIMMS) schedule targeting the inguinal, axial, brachial, and popliteal lymph nodes. Lymph nodes were harvested, non B cells depleted using a B cell isolation kit (STEMCELL) and fused to P3X.653 myeloma cells to generate hybridomas. 384-well based ELISA screens utilizing soluble CD16b, Clec9a-Fc, CD16a (AcroBiosystems) or KLRG1-Fc (R&D) biotinylated with NHS-PEG12-biotin (Thermo) were completed 12 days after hybridoma fusion. Primary ELISA hits were subsequently screened in mixed-culture flow cytometry binding assays. For CD16b specific binders, K562 cells were transfected with CD16b and loaded with 1uM CMFDA (Thermo #C2925) following manufacturer protocols. These cells were then mixed in the same well with WT K562 cells transfected with CD16a. For Clec9a binders, K562 cells were transfected with Clec9a and mixed together with CMFDA dye loaded WT K562 cells. Hybridoma supernatants were added to the appropriate antigen expressing cell wells, incubated, washed, and detected with a polyclonal Goat anti-mouse IgG Fc-specific AF 647 conjugate (Jackson) by flow cytometry (BD Fortessa). Results were analyzed using FlowJo software to generate MFI values and plotted using GraphPad Prism

### 4.4 VH and VL recovery

Hybridomas were sequenced using Mouse BCR profiling kit (Takara) and cDNA was sequenced using next generation sequencing (Azenta).

### 4.5 Surface-molecule quantification

Quantification of CD33 levels on the surface was done using QIFIKIT quantitative analysis kit (Agilent) with the CD33 antibody clone WM53 (BD Biosciences). The protocol was previously described (25, 27).

### 4.6 Jurkat cell reporter assay

The Jurkat functional assay was performed as previously described (72). Briefly, NFAT-luciferase reporter Jurkat cells were harvested and washed in PBS and transfected with DNA plasmids using the Neon transfection system for 100 µL reactions (# MPK10096). Electroporation conditions were set to 3 pulses at 1500V for 10 ms. Cells were rested overnight in 1 mL of media in 24 well plates. The next day Jurkat cells were combined with target cells and cocultured for 6 hrs before the ONE™ step luciferase firefly assay system (BPS Bioscience) was used to determine luminescence intensity on a Tecan Infinite M1000. For mRNA titration experiments, target cells were either HeLa or K562 cells that were also transfected one day before with mRNA titration using the 4D nucleofector (Lonza). For HSC cell titration experiments, target human CD34(+) HSPCs were thawed, rested overnight, collected, counted, and seeded ranging from 675 to 15,000 cells/well in a 384-well plate in a 2-fold dilution series.

### 4.7 Primary T cell engineering

Human PBMCs or enriched T cells were thawed into warm T cell media, washed and resuspended in T cell media supplemented with recombinant human IL-2 and stimulated with TransAct (Miltenyi Biotec) at 1:100 titer according to manufacturer recommendations. T cells were plated into a 24 well plate at 1 million/mL in 2 mL/well for 48 hrs. After 48 hrs, cells were harvested, washed with PBS and resuspended in Lonza P3 buffer (# V4XP-3032) at 20 μL/reaction. 1 μg of PiggyBac transposase mRNA (Hera Biolabs) and 1.6 μg of transposon plasmid DNA were added to 1 million T cells which were then electroporated using the 4D nucleofector (Lonza) program EO-115 in 16-well cuvette strip. Cells were immediately transferred to 200 μL of warm media in a 96 well round-bottom plate and cultured overnight, re-stimulated with TransAct at 1:100 titer, then transferred to 500 μL in 48 well plates. After 1-3 days, cells were transferred to GREX24 (Wilson Wolf) for expansion until use in assays. For the *in vivo* study, transgene positive cells were enriched with puromycin selection (0.5ug/mL) beginning 4 days after transfection.

### 4.8 Target cell engineering

K562 cells were transfected with transposon plasmids encoding CD33 and CD16b in the presence of transposase mRNA to establish stable cell lines. Two million K562 cells, 1.6 μg of DNA plasmid and (0.125-1) μg of transposase mRNA were combined in 20 μL of Lonza SF buffer (# V4XC-2032) and then electroporated using the 4D nucleofector (Lonza) program FF-120 in 16-well cuvette strip. Cells were transferred to warm media in a 12-well plate for recovery. CD16b+ MV-4-11 cells were created by lentiviral transduction followed by cell sorting using a FACSMelody (BD). CD33 and CD16b expression was confirmed by flow cytometry using CD33 antibody clone WM53 (BD Biosciences) and CD16 antibody clone 3G8 (BD Biosciences). GFP-firefly luciferase or GFP-renilla luciferase was introduced to engineered MV-4-11 or engineered K562, respectively, via lentiviral transduction followed by cell sorting using a FACSMelody. To generate B2M-, SPN-, or CD33- variants of cell lines, genetic modification using CRISPR-Cas9 was performed. Streptococcus pyogenes HiFi Cas9 protein (IDT) were mixed at 1:3 molar ratio to form ribonucleoprotein complex then transfected into desired cell lines using 4D-Nucleofector (Lonza). Knockout cells were enriched by cell sorting using a FACSMelody using appropriate antibodies (B2M: W6/32, SPN: 1G10, CD33: WM53).

### 4.9 Cytotoxicity assays

Cytotoxicity was assessed via imaging on the IXM system (Molecular Devices) and endpoint luciferase activity was recorded as a secondary measurement (26). Target cells, K562 or MV-4-11, were plated in a 384-well plate with transparent bottom coated with poly-D-Lysine (Greiner #781946) in T cell media and allowed to adhere for 1 hr. Engineered T cells were profiled via flow cytometry for construct expression using recombinant human CD33 (Acro Biosystems) and recombinant human CD16b (NA2) (Acro Biosystems). T cells were normalized to a single transgene-positive percentage by addition of untransposed cells. T cells were washed with PBS, resuspended in T cell media without IL-2, and combined with the target cells at a titration of effector to target (E:T) cell ratios.

### 4.10 Mouse *in vivo* assay

NSG-SGM3 mice were purchased from Jackson Laboratories and housed at the Charles River Accelerator and Development Lab. Post acclimation, mice were injected with 2 million target cells in 100uL via tail vein. MV-4-11 target cells were harvested during log-phase growth, washed with HBSS and resuspended in HBSS at 2 million cells/100 μL. Six days later, engineered T cells were injected at 7.5 million cells per mouse in 200 μL of HBSS. *In vivo* Bioluminescence imaging (BLI) was performed twice a week. Briefly, 150 μL of D-luciferin (150 μg/ml, Perkin Elmer) was injected intraperitoneally into each animal. After 15 minutes, and additional time points, mice were imaged belly up. At the end of the study femur and tibia bones were collected and bone marrow was extracted from one bone per mouse for flow cytometry analysis. The following antibodies were used to profile bone marrow by flow cytometry: anti-mouse CD45-BV421 (Biolegend), anti-human CD8-PerCP-Cy5.5 (Biolegend), anti-human CD4-PE-Cy7 (Biolegend) and anti-human CD3-APC-e780 (Thermo Fisher). Recombinant human CD33 and CD16b were also included to stain transgene-positive T cells. Xenograft MV-4-11 cells were measured via GFP fluorescence.

### 4.11 Statistical analysis

Statistical analyses were performed using GraphPad Prism 10.1. Data from Jurkatcell-based experiments are shown as mean ± standard deviation of technical replicates. Curve fitting was performed using four-parameter non-linear regression analysis, with EC50 and IC50 values calculated directly from best-fit curves. Maximum % inhibition values are calculated from interpolated values of these best-fit curves at the top blocker mRNA titration point. All Jurkat assay summary metrics are reported in **Supplementary Table 2**. *In vivo* data were analyzed using a Kruskal-Wallis *H* test followed by a *post hoc* Dunn’s multiple comparisons test to correct for multiple comparisons; adjusted p-values were reported and the null hypothesis was rejected for adjusted p <0.05.

## Data Availability

The data presented in the study are deposited in the GenBank reposity, accession numbers PV220986-92.
