# Supplementary material for: Multi-targeted, NOT gated CAR-T cells as a strategy to protect normal lineages for blood cancer therapy
Source: Front Immunol. 2025 Mar 21;16:1493329. doi: 10.3389/fimmu.2025.1493329 (PMC11968376; doi:10.3389/fimmu.2025.1493329)
Supplement: Supplementary file 1 [file DataSheet1.pdf]

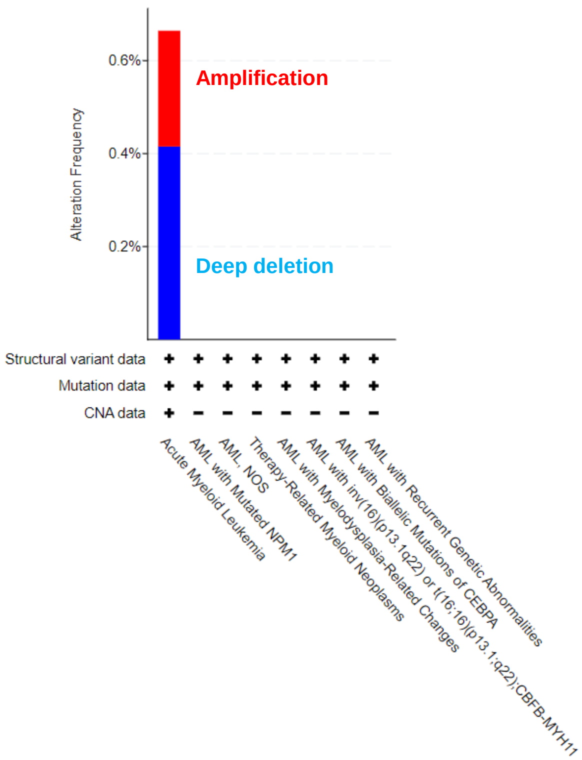

Supplemental Figure 1. FCGR3B (CD16B) downregulation is not the consequence of genetic deletion.
Figure from cBioportal.org illustrating low genomic alteration of the FCGR3B in different sample data sets.

A

Sensitivity profiling to CD33 antigen

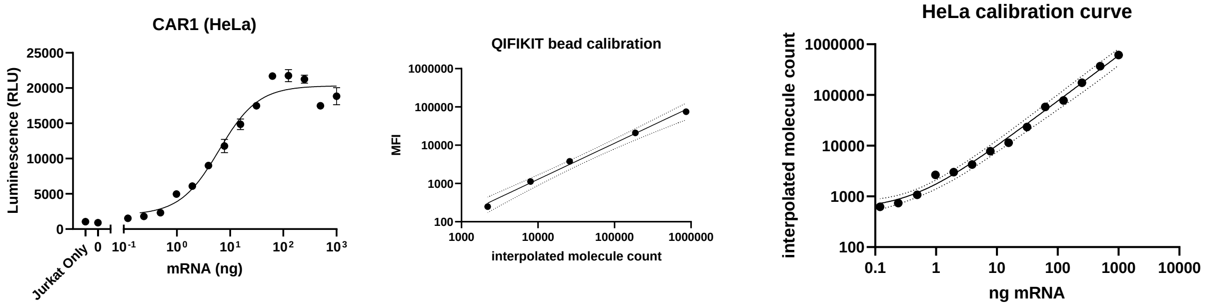

CD33 CARs	HeLa EC50 (molecules)	K562 EC50 (molecules)
CAR1	13,600 (n=3)	10,500 (n=2)
CAR2	8,000 (n=2)	7,500 (n=1)
CAR3	4,500 (n=2)	9,000 (n=3)
CAR4	1,800 (n=2)	2,200 (n=2)

B

Surface CD33 molecule levels in AML cell lines

Cell line	CD33 molecules/cell
MOLM-13	15,000
THP-1	33,000
MV-4-11	10,000
HL-60	14,000

Supplemental Figure 2. CD33 CARs can recognize relevant levels of CD33.
(A) Jurkat reporter cell line activation assay with representative data from CAR1. Jurkat cells were transfected with CAR DNA plasmids and cocultured with target cells (HeLa or K562 CD33 KO) transfected with a titration of the CD33 mRNA. Firefly luciferase was measured 6 hours post coculture start. EC50 values were calculated in units of ng of mRNA and then converted to molecules using QIFIKIT. (B) Table showing CD33 molecule levels on the cells surface of commonly used blood cancer cell lines.

A

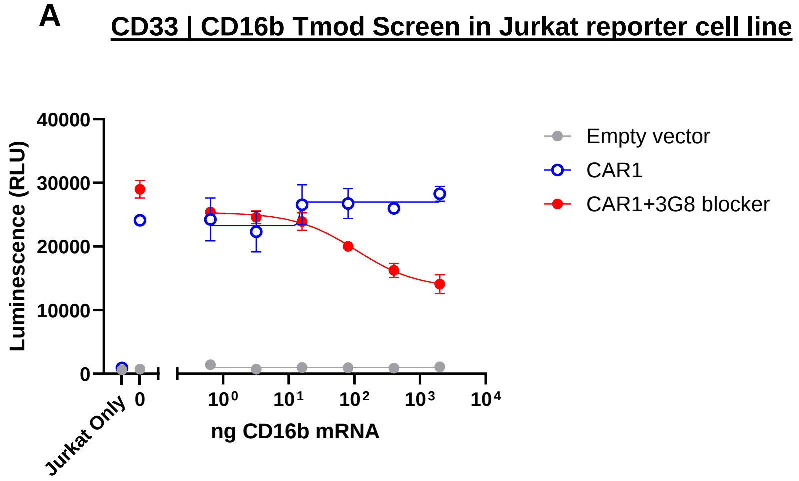

B

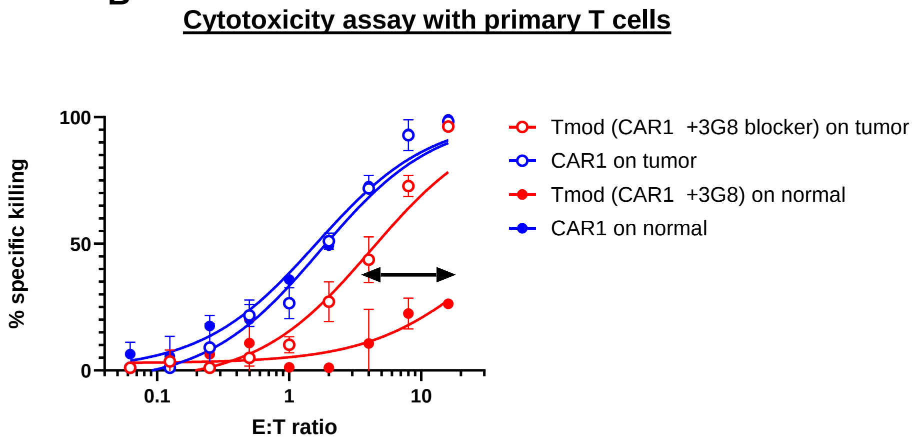

Supplemental Figure 3. Commercially available CD16 antibody shows ligand dependent inhibition when converted into Tmod format.
(A) Jurkat reporter cell assay. Jurkat cells are transfected with two plasmids, one encoding the CAR and the other a blocker. Jurkat cells were then cocultured with target cells transfected with a titration of CD16b mRNA. (B) Tmod T cells were cocultured with target cells for 48 hours and killing measured via luciferase signal at the end. Target cells are K562s that over express either CD33 to model the tumor or both CD33 and CD16b to model the normal tissue.

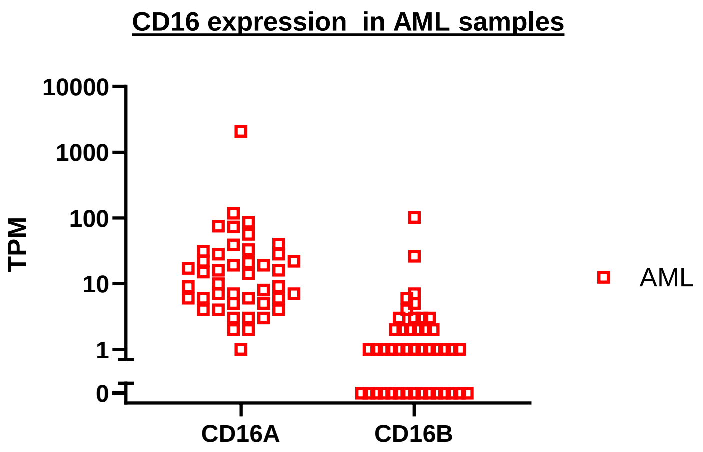

Supplemental Figure 4. FCGR3A (CD16a) expression in AML samples is higher than FCGR3B (CD16b) levels.
RNA-seq expression data from primary AML samples retrieved from the Ley Lab (https://leylab.shinyapps.io/TCGA_AML_Web_App/).

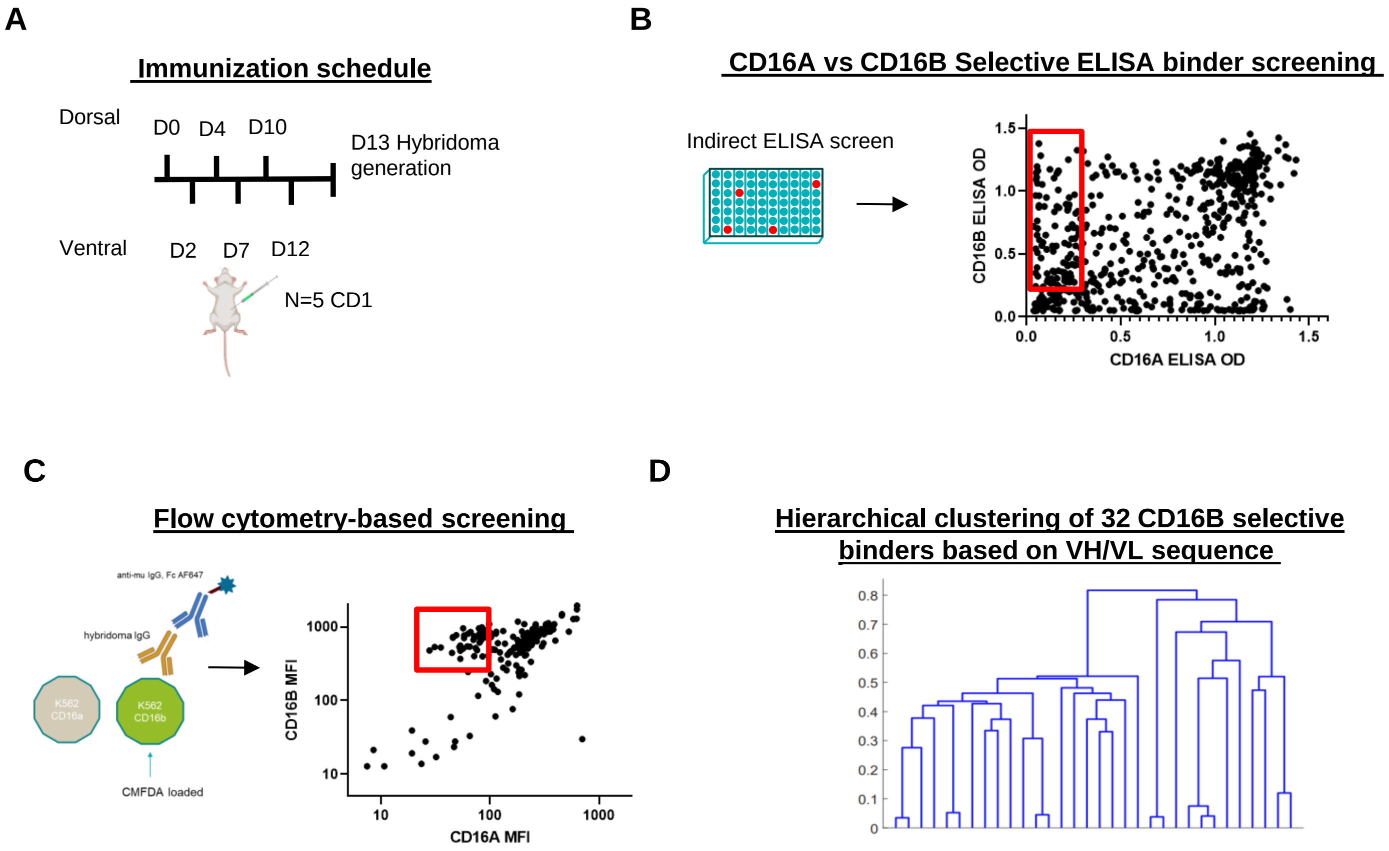

Supplemental Figure 5. Identification of hybridoma-derived CD16b-specific binders. A) Mouse immunization schedule. B) CD16b-specific binders were identified by indirect ELISA screens using biotinylated CD16a or CD16b soluble protein. Wells with CD16b-selective binding (red square) were selected for further analysis. C) Multiplexed FACS-based CD16a vs CD16b binding screens utilized CMFDA-loaded K562 cells overexpressing CD16b mixed with non-colored K562 overexpressing CD16a to confirm selective binding to the membrane-bound form of CD16b. D) Dendrogram showing diversity of 32 clones chosen for blocker generation. Hamming distance (y-axis) of 0.1 represents approximately 10 residue differences.

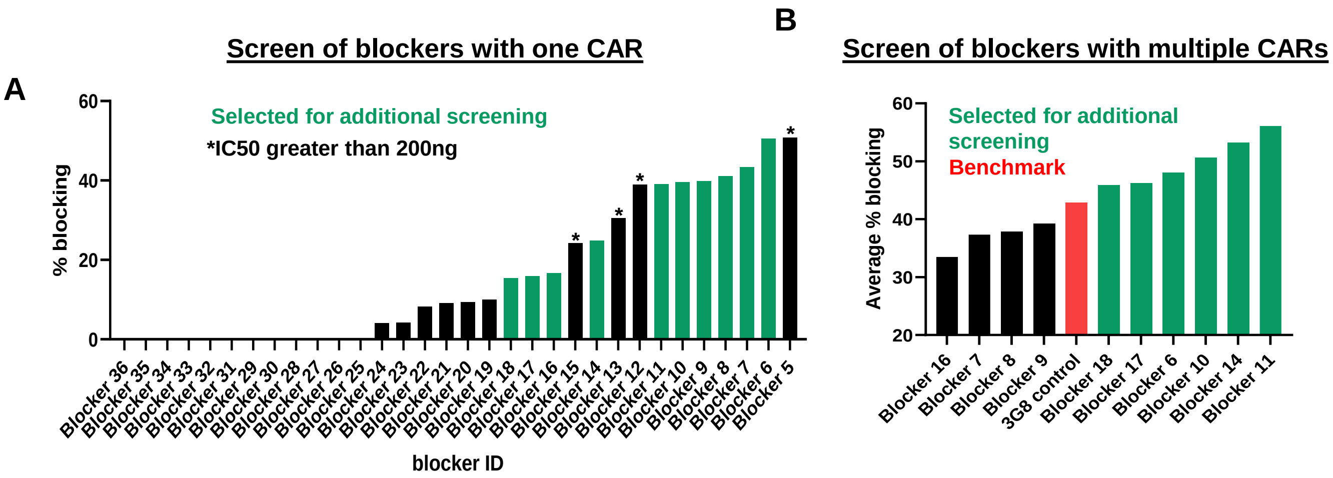

C

Blockers

IC50 (ng mRNA)

Activators	11	18	14	10	6	17	3G8
CAR2	**8.3**	**233.1**	114.6	48.7	**42.7**	**39.9**	89.4
CAR3	**318.4**	**-**	15.7	66.7	**48.9**	**33.2**	26.8
CAR4	0.3	15.4	3.2	5.5	43.0	**17.9**	15.3

% Block

Activators	11	18	14	10	6	17	3G8
CAR2	**76**	**69**	64	69	**70**	**63**	47
CAR3	**51**	**-**	68	75	**69**	**72**	32
CAR4	25	64	72	73	74	**79**	60

Supplemental Figure 6. CD33/CD16B Tmod screening in Jurkat cells. (A) Rank-ordered inhibition by blockers using a single CD33 CAR. (B) Rank-ordered inhibition using the average percent blocking with 4 different CD33 CARs. (C) Summary metrics showing IC50 and maximum percent block from mRNA titration experiments shown in Figure 2C. The 9 combinations carried forward to primary T experiments are shown in blue font.

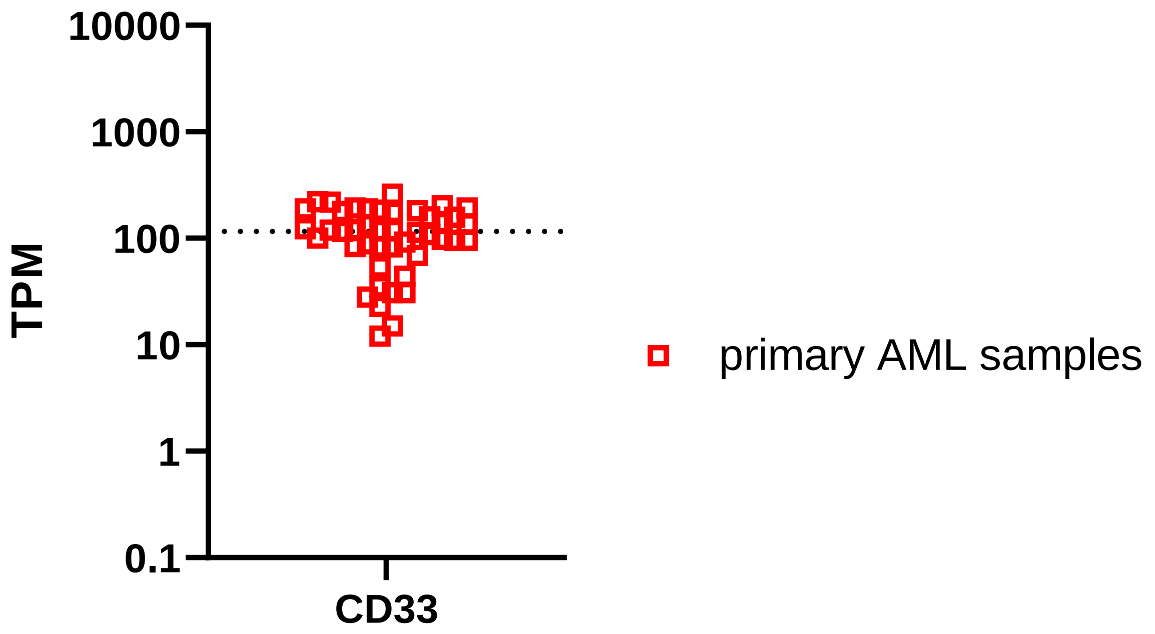

Supplemental Figure 7. CD33 gene expression in MV-4-11 matches AML primary samples.
CD33 gene expression in primary AML samples retrieved from the Ley lab data set. The level of CD33 in MV-4-11 (from DepMap) is plotted as dashed line.

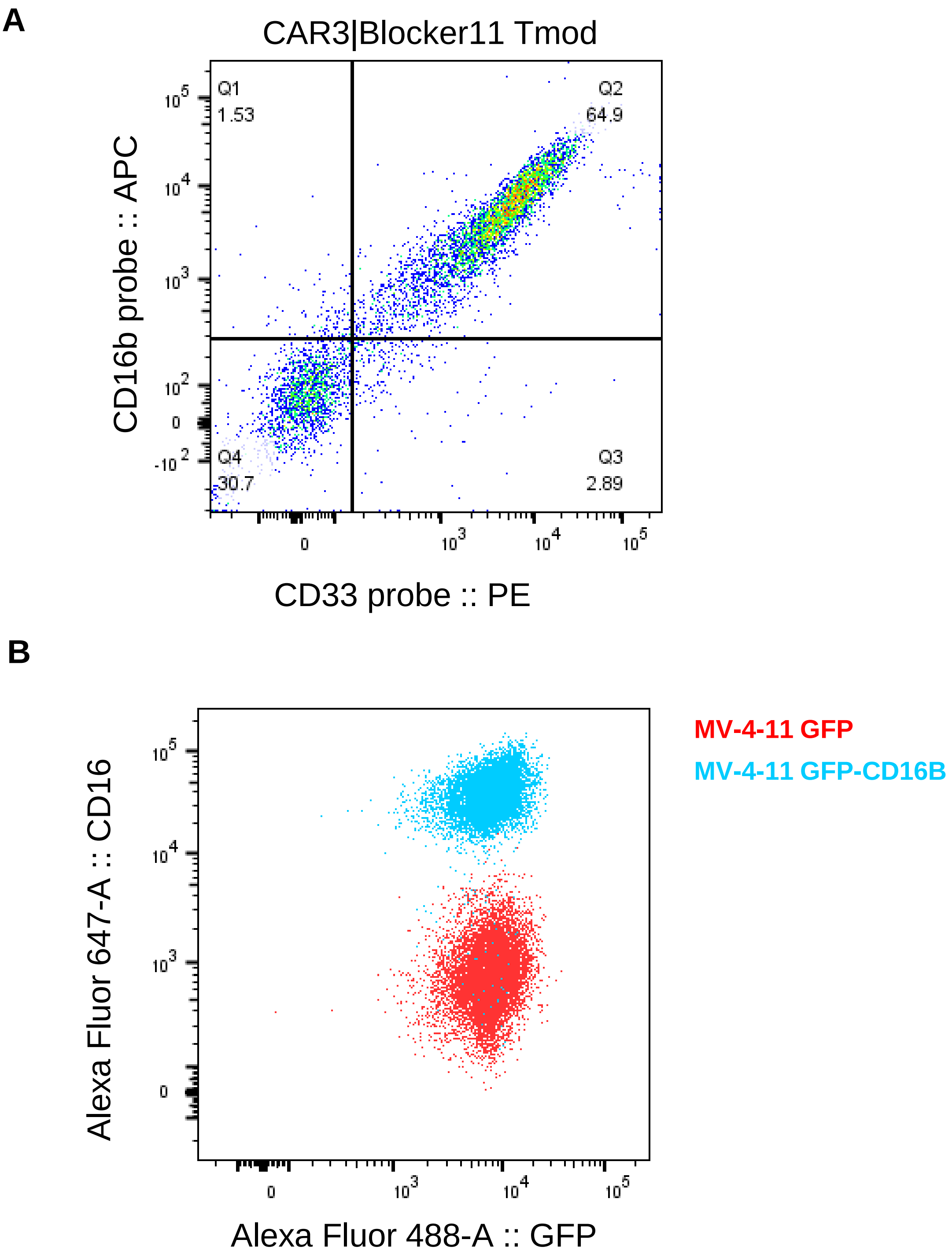

Supplemental Figure 8. Characterization of T cells and target cells for cytotoxicity experiments. (A) Representative flow cytometry data of Tmod expression in primary T cells (B) Flow cytometry-based analysis of CD16b and GFP expression in the engineered MV-4-11 cell lines.

A

ID	Membrane distal	Membrane proximal	Tandem format
1	CD33(CAR3)	SPN(CAR1)	■LHHL
2	CD33(CAR3)	SPN(CAR1)	●HLLH
3	SPN(CAR1)	CD33(CAR3)	■LHHL
4	SPN(CAR1)	CD33(CAR3)	●HLLH
5	CD33(CAR1)	SPN(CAR1)	■LHHL
6	CD33(CAR1)	SPN(CAR1)	●HLLH

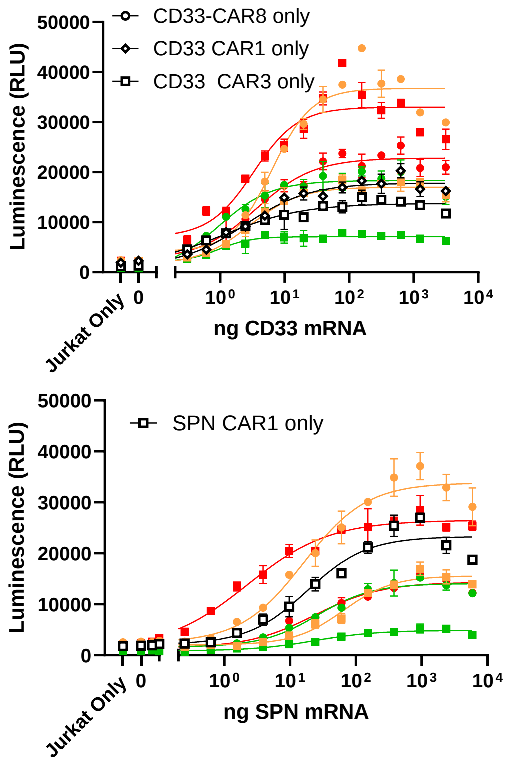

B

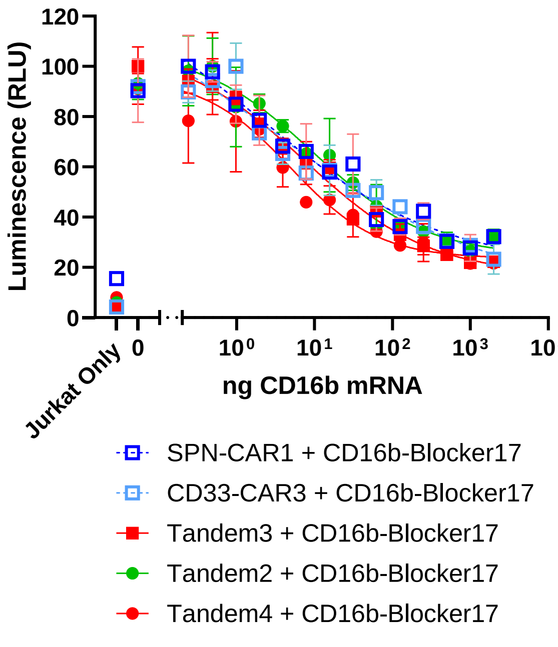

Supplemental Figure 9. SPN-CD33 tandem CAR screening. (A) 6 SPN-CD33 tandem variants screened in Jurkat cell functional assay with either SPN or CD33 mRNA titration. (B) Top 3 tandem CD33-SPN CARs blocked by CD16b blocker in Jurkat cell functional assay.

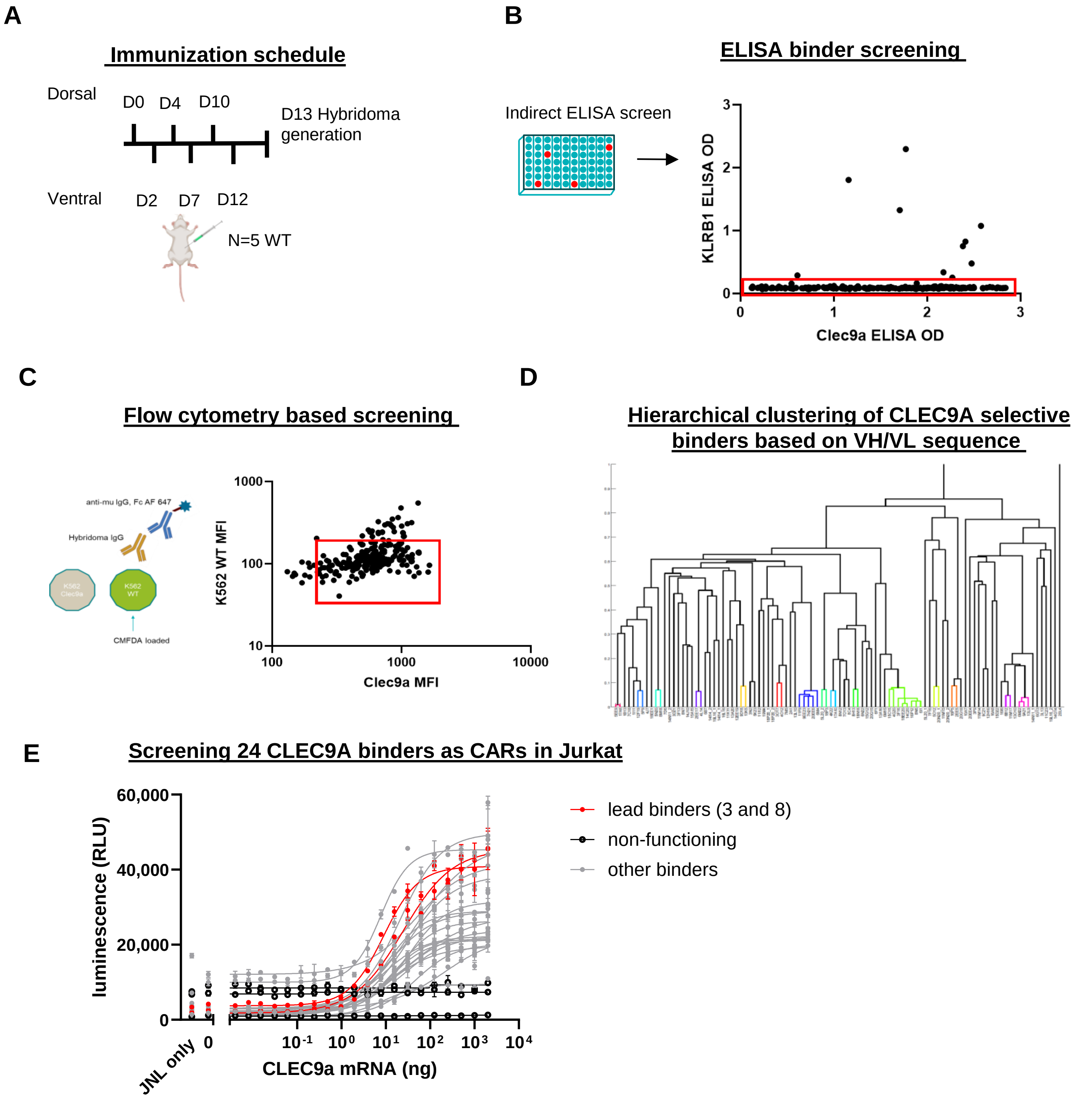

Supplemental Figure 10. Identification of hybridoma-derived Clec9a-specific binders. **(**A) Mouse immunization schedule. B) Clec9a specific binders were identified by indirect ELISA screens using biotinylated huClec9a-Fc and huCD161-Fc (KLRB1) extracellular domain proteins. Wells with Clec9a-selective binding (red square) were selected for further analysis. C) Multiplexed FACS binding assays were performed on either huClec9a(+) (overexpressed) or huClec9a(-) K562 (parent) cells. Values represent median fluorescence intensity (MFI) after detection with appropriate secondary antibody to confirm selective binding to the membrane bound form Clec9a. D) Dendrogram showing diversity of clones identified. (E) Jurkat cell functional readout of 24 Clec9a binders cloned as CARs.

Cell type	RNAseq format	n	Source	Data source link
AML	bulk	178	TCGA LAML	DOI: 10.1056/NEJMoa1301689
	bulk	187	Ped TARGET, phs000465	https://portal.gdc.cancer.gov
	SC	35	VanGalen2019	https://www.ncbi.nlm.nih.gov/geo/query/acc.cgi?acc=GSE116256
MM	bulk	859	MMRF-CoMMpass	https://portal.gdc.cancer.gov/projects/MMRF-COMMPASS
NHL	bulk	481	NCICCR-DLBCL	https://portal.gdc.cancer.gov/projects/NCICCR-DLBCL
		10	CLL (GSE128668)	https://www.ncbi.nlm.nih.gov/geo/query/acc.cgi?acc=GSE128668
		311	CLL-ICGC	https://dcc.icgc.org/releases/current/Projects/CLLE-ES
HSC	SC	4	Kaufman2021	https://www.ncbi.nlm.nih.gov/geo/query/acc.cgi?acc=GSE148884
	SC	2	VanGalen2019	https://www.ncbi.nlm.nih.gov/geo/query/acc.cgi?acc=GSE116256
	bulk	3	EPCR	https://www.ncbi.nlm.nih.gov/geo/query/acc.cgi?acc=GSE77128
	bulk	5	JMML	https://www.ncbi.nlm.nih.gov/geo/query/acc.cgi?acc=GSE183252
	bulk	7	ITGA	https://www.ncbi.nlm.nih.gov/geo/query/acc.cgi?acc=GSE130974
	bulk	6	Blueprint	https://ega-archive.org/datasets/EGAD00001002316
Monocytes	bulk	12	Monaco	DOI: 10.1016/j.celrep.2019.01.041
Neutrophils	bulk	4	Monaco	DOI: 10.1016/j.celrep.2019.01.041
T cells	bulk	3	Ley Lab	https://proteomics.leylab.org/

Supplemental Table 1. Data sources for target mRNA expression. For datasets from single-cell (SC) RNAseq datasets, each point represents a pseudobulked sample. For pseudobulking, expression values (counts) from a group of cells belonging to a given cell type (e.g. HSC or AML) from the same donor were aggregated.

Fig 4A

	EC50 (ng)	
	CD19 mRNA	CD20 mRNA
CD19-CAR	5.5	-
CD20-CAR	-	3.4
CD19-CD20-CAR	2.3	3.4

		IC50 (ng A*02 mRNA)			Max % Block		
		+CD19	+CD20	+CD19+CD20	+CD19	+CD20	+CD19+CD20
CD19-CAR	A*02 Blocker	32.5		145.6	61.9		66.3
CD20-CAR	A*02 Blocker		26.5	24.9		67.5	65.9
CD19-CD20-CAR	A*02 Blocker	9.6	11.1	21.6	73.2	76.5	77.3

Fig 4B

		IC50 (ng)		Max % Block	
		A*02 mRNA	A*03 mRNA	A*02 mRNA	A*03 mRNA
MSLN-CAR	A*03 Blocker	-	3.7	-	82.4
MSLN-CAR	A*02 Blocker	12.6	-	73.7	-
MSLN-CAR	A*02/A*03 Blocker	18.7	13.2	64.4	70.8
MSLN-CAR	A*03/A*02 Blocker	44.2	25.9	43.4	65.7

Figure 4C

		IC50 (ng)		Max % Block	
		A*02 mRNA	A*03 mRNA	A*02 mRNA	A*03 mRNA
CD19-CAR	A*02-Blocker	19.7	-	82.4	-
CD20-CAR	A*02-Blocker	7.2	-	75.2	-
CD19/CD20-CAR	A*02-Blocker	6.1	-	72.0	-
CD19-CAR	A*03-Blocker	-	4.5	-	56.1
CD20-CAR	A*03-Blocker	-	2.3	-	71.5
CD19/CD20-CAR	A*03-Blocker	-	2.7	-	77.6
CD19-CAR	A*02/A*03-Blocker	35.8	10.9	54.9	60.4
CD20-CAR	A*02/A*03-Blocker	19.4	6.2	67.6	64.0
CD19/CD20-CAR	A*02/A*03-Blocker	16.6	3.8	72.6	71.8

Fig 6D

		Max % Block
CAR1	Blocker 6	71.1
CAR1	Blocker 17	55.2
CAR1	Blocker 9	55.1
CAR1	3G8	62.2
CAR2	Blocker 11	73.9
CAR2	Blocker 6	69.3
CAR2	Blocker 17	65.5
CAR2	Blocker 9	57.8
CAR2	3G8	61.9

Fig 6E

	EC50 (ng)	
	CD33 mRNA	SPN mRNA
CD33 CAR	1.1	
SPN CAR		18.3
CD33/SPN CAR	3.6	23.8

		IC50 (ng)	
		CD16b mRNA	Max % Block
CD33 CAR	CD16b Blocker	11.9	72.7
SPN CAR	CD16b Blocker	10.5	69.7
CD33/SPN CAR	CD16b Blocker	5.4	75.4

Fig 6G

		Max % Block	
		CLEC9A mRNA	CD16b mRNA
CD33 CAR	CLEC9A Blocker	49.5	
CD33 CAR	CD16b Blocker		86.1
CD33 CAR	CLEC9A/CD16b Blocker	24.3	70.0

Fig 6H

		EC50 (ng)		IC50 (ng)		Max % Block	
		CD33 mRNA	SPN mRNA	CD16b mRNA	CLEC9A mRNA	CD16b mRNA	CLEC9A mRNA
CD33 CAR	CD16b Blocker	14.0		8.3		68.4	
SPN CAR	CD16b Blocker		53.5	15.5		59.6	
CD33 CAR	CLEC9A Blocker	12.4			5.9		51.6
SPN CAR	CLEC9A Blocker		76.6		8.8		39.4
CD33/SPN CAR	CD16b Blocker	6.3	51.2	7.0		62.9	
CD33/SPN CAR	CLEC9A Blocker	5.1	104.7		4.7		44.1
CD33/SPN CAR	CLEC9A/CD16b Blocker	4.5	63.2	6.6	28.6	55.7	41.5

Supplemental Table 2. Summary metrics for Jurkat blocking curves.
Shows Metrics summarizing main figures as annotated
